# Supplementary material for: Relative Importance of Soluble and Microsomal Epoxide Hydrolases for the Hydrolysis of Epoxy-Fatty Acids in Human Tissues
Source: Int J Mol Sci. 2021 May 8;22(9):4993. doi: 10.3390/ijms22094993 (PMC8125816; doi:10.3390/ijms22094993)

Relative importance of soluble and microsomal epoxide hydrolases for the hydrolysis of epoxy-fatty acids in human tissues (Supplemental Material)

Christophe Morisseau,[1] Sean D. Kodani,[1] S. George Kamita,[1] Jun Yang,[1] Kin Sing Stephen Lee,[1] and Bruce D. Hammock[1*]

[1] Department of Entomology and Nematology, and U.C. Davis Comprehensive Cancer Center, University of California Davis, Davis, CA 95616 USA

* Correspondence: bdhammock@ucdavis.edu; Tel.: +1 530 752 7519

Table S1: Information regarding the origins of the human tissue extracts

Vendor	Tissue	Donor sex	Donor age	[protein] mg/mL	Lot #	buffer
Sekisui XenoTech, LLC 1101 W Cambridge Cir Dr, Kansas City, KS 66103 https://www.xenotech.com/	Kidney S9	Mix of 13		4	1010324	Tris-HCl, (50 mM pH 7.4),150 mM KCl, 1 mM EDTA, 20% Glycerol, Heparin, PMSF, Leupeptin, DTT & Aprotinin
	Liver S9	Mix of 50		20	1210091	
	Lung S9 non-smoker	Mix of 4		5	1410245	
	Lung S9 smoker	Mix of 5		5	1110009	
	Small Intestine S9	Mix of 10		5	1410073	
BioChain Institute Inc. 39600 Eureka Drive Newark, CA 94560 https://www.biochain.com/	Adrenal	M	25	3	A304029	HEPES (50mM pH 7.9), containing $MgCl_2$, KCl, EDTA, sucrose, glycerol and cocktail of protease inhibitor
	Esophagus	M	77	5	B906502	
	Heart	F	76	3	B109096	
	Hippocampus	M	66	3	A805194	
	Kidney	F	83	3	A508132	
	Liver	M	26	3	A512343	
	Lung	M	50	3	B406015	
	Ovary	F	45	3	A603194	
	Pancreas	F	76	3	B110220	
	Skin	F	40	2.67	A410143	
	Small intestine	F	68	3	A302181	
	spleen	M	24	3	A804197	
	Stomach	F	66	5	B906501	
	Testis	M	56	3	A301192	
	Tongue	M	71	5	B906503	

Table S2: Concentrations of sEH and mEH in human tissue extracts on the basis of western blot analysis and selective activity.

	**[sEH] (nM)**			**[mEH] (nM)**	
Tissue	Western	t-DPPO		Western	c-SO
**Adrenal**	< 19	14.1 ± 1.6		90 ± 42	287 ± 52
**Esophagus**	< 8	4.7 ± 0.4		13	12.5 ± 0.4
**Heart**	6 ± 1	12.9 ± 0.4		5	10 ± 4
**Hippocampus**	5 ± 1	4.8 ± 0.4		7	17 ± 2
**Kidney**	< 19	41.6 ± 1.2		< 23	22 ± 11
**Liver**	41 ± 6	88.2 ± 2.7		198 ± 114	850 ± 52
**Lung**	< 19	1.8 ± 0.4		< 23	20 ± 2
**Ovary**	< 4	1.2 ± 0.2		6	5.8 ± 0.8
**Pancreas**	39 ± 18	22.5 ± 3.2		108 ± 18	67 ± 4
**Skin**	< 16.7	3.4 ± 1.0		< 21	6 ± 2
**Small Intestine**	13 ± 3	12.4 ± 0.4		10 ± 2	24 ± 2
**Spleen**	5	3.2 ± 0.8		< 5	5.8 ± 1.0
**Stomach**	< 5	1.6 ± 0.5		12	12 ± 2
**Testis**	13 ± 6	5.8 ± 0.4		132 ± 18	96 ± 7
**Tongue**	24 ± 11	9.6 ± 0.8		14	12 ± 1

Results are average ± standard deviation (n = 3).

Table S3: Hydrolytic activity of human tissue extracts for 14,15-EET and 13,14-EDP each at 5 μM.

	tissue	Specific activity ($nmol.min^{-1}.mg^{-1}$)	
		14,15-EET	13,14-EDP
S-9	kidney	0.88 ± 0.08	1.22 ± 0.09
	liver	2.14 ± 0.36	2.7 ± 0.2
	lung non-smoker	0.098 ± 0.006	0.068 ± 0.002
	lung smoker	0.083 ± 0.006	0.089 ± 0.007
	intestine	1.3 ± 0.3	1.96 ± 0.03
Whole cell extract	Adrenal	0.23 ± 0.03	0.10 ± 0.01
	Esophagus	0.06 ± 0.02	0.009 ± 0.001
	Heart	0.18 ± 0.05	0.073 ± 0.003
	Hippocampus	0.07 ± 0.02	0.012 ± 0.007
	Kidney	0.8 ± 0.1	0.46 ± 0.02
	Liver	1.5 ± 0.2	1.8 ± 0.1
	Lung	0.06 ± 0.01	0.016 ± 0.003
	Ovary	0.020 ± 0.003	0.010 ± 0.001
	Pancreas	0.58 ± 0.04	0.12 ± 0.01
	Skin	0.06 ± 0.02	0.003 ± 0.001
	Small Intestine	0.26 ± 0.03	0.11 ± 0.01
	Spleen	0.04 ± 0.01	0.005 ± 0.002
	Stomach	0.016 ± 0.003	0.010 ± 0.001
	Testis	0.10 ± 0.03	0.028 ± 0.003
	Tongue	0.10 ± 0.03	0.019 ± 0.001

Results are average ± standard deviation (n = 3).

Table S4: Gene accession numbers.

Protein name	Gene name	Gen-Bank Accession number
Epoxide hydrolase 3	EPHX3 or ABHD9	BC132960
Epoxide hydrolase 4	EPHX4 or ABHD7	BC041475

Figure S1: Western blot analysis of human tissue extracts

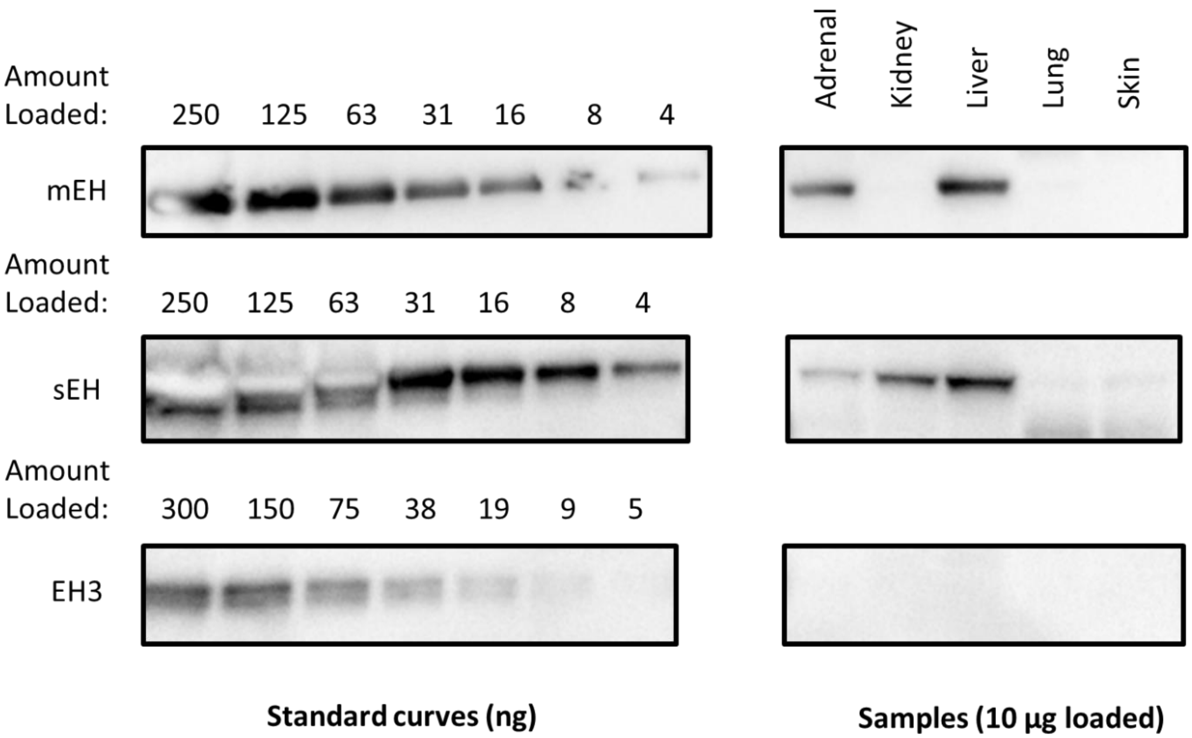

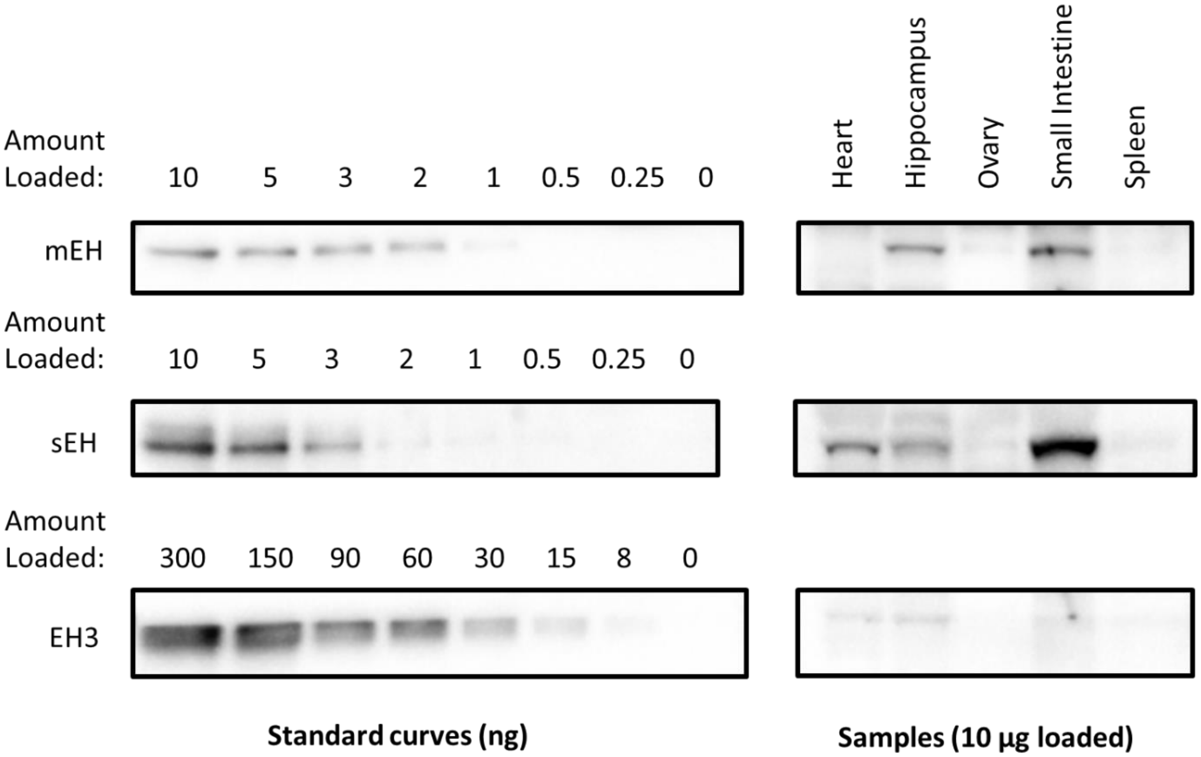

Figure S2: Hydrolysis selectivity of the human tissue extracts for EpFAs

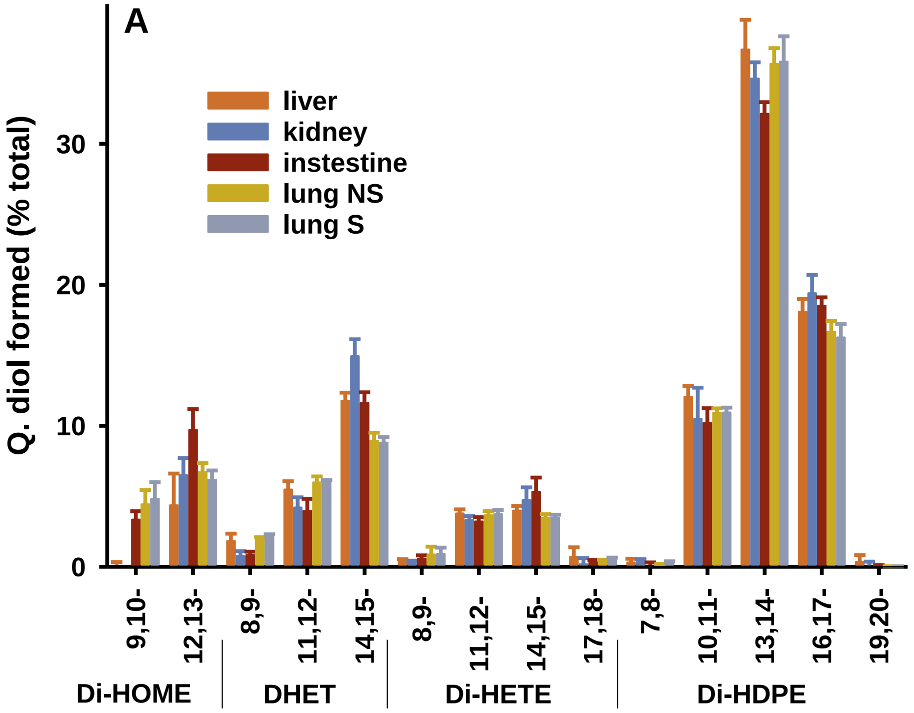

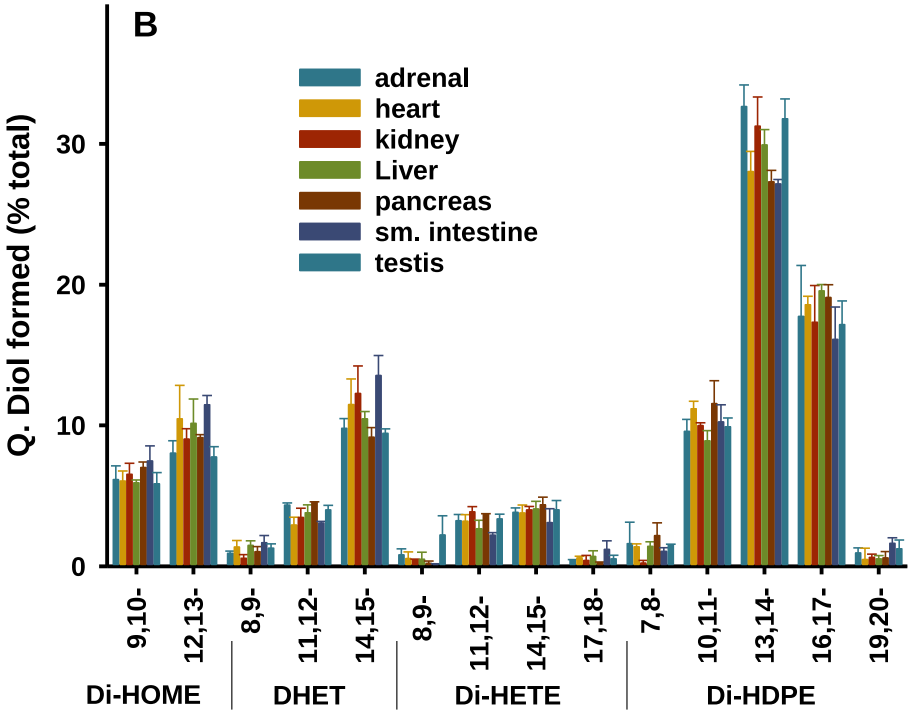

Figure S3: SDS-PAGE analysis of recombinant EHs. All proteins were produced in High Five cells (Invitrogen) that were grown at 27°C in ESF921 medium. The recombinant proteins were prepared as described in section 4.3 of the paper. The proteins were revealed using Coomassie blue. Identity of the targeted proteins was confirmed by Western-blot (not shown). Scanning densitometry suggests that the human sEH purity is >90%, the human mEH purity is 80%, the human EH3 purity is around 5%, and the human EH4 purity around 8%.

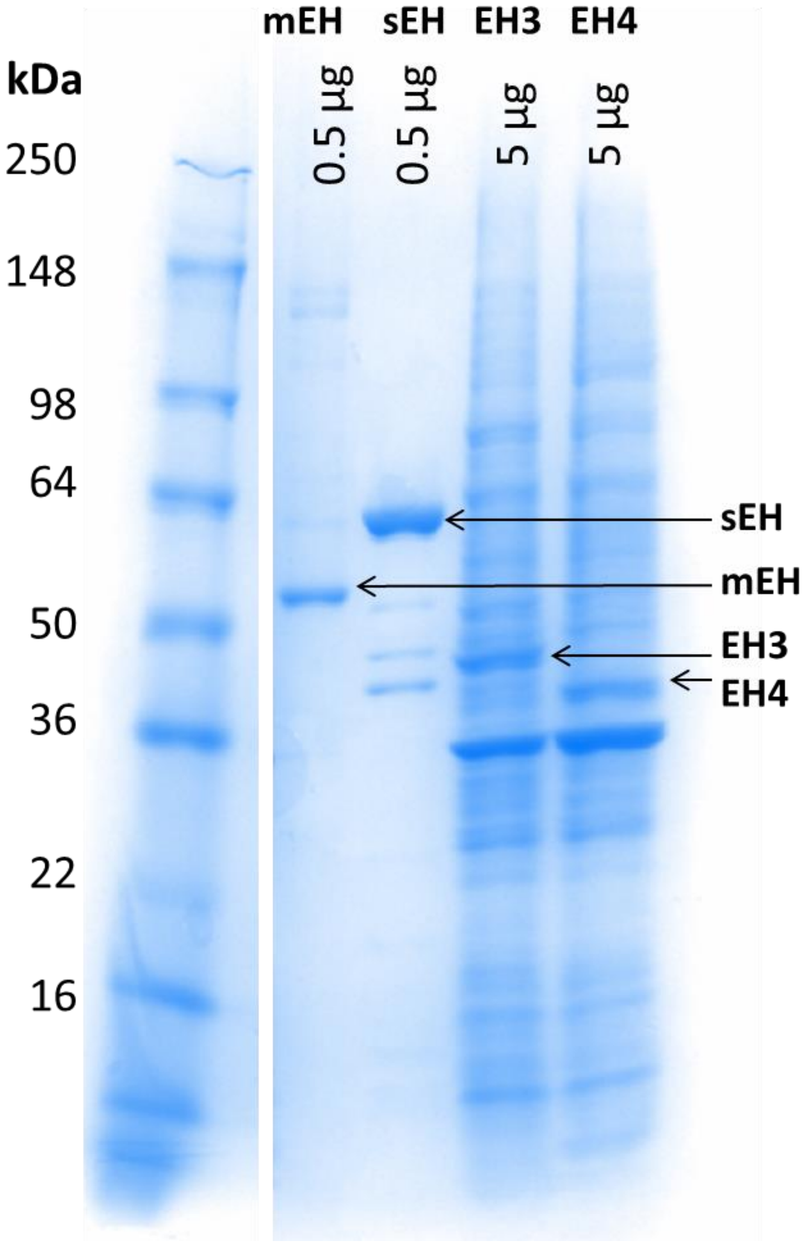

Supplement: Supplementary file 1 [file ijms-22-04993-s001.zip › ijms-1209212-supplementary.pdf]
